# Probiotic potential of *Tetragenococcus halophilus* EFEL7002 isolated from Korean soy Meju

**DOI:** 10.1186/s12866-022-02561-7

**Published:** 2022-06-06

**Authors:** Da Hye Kim, Seul-Ah Kim, Yu Mi Jo, Hee Seo, Ga Yun Kim, Seong Won Cheon, Su Hwi Yang, Che Ok Jeon, Nam Soo Han

**Affiliations:** 1 Brain Korea 21 Center for Bio-Health Industry, Division of Animal, Horticultural, and Food Sciences, Chungbuk National University, Cheongju, 28644 Republic of Korea; 2 Department of Life Science, Chung-Ang University, Seoul, 156-756 Republic of Korea

**Keywords:** *Tetragenococcus halophilus*, Probiotics, Biogenic amine, Salty fermented foods

## Abstract

**Background:**

Probiotic starters can improve the flavor profile, texture, and health-promoting properties of fermented foods. *Tetragenococcus halophilus* is a halophilic lactic acid bacterium that is a candidate starter for high-salt fermented foods. However, the species is known to produce biogenic amines, which are associated with neurotoxicity. Here, we report a probiotic starter strain of *T. halophilus*, EFEL7002, that is suitable for use in high-salt fermentation.

**Results:**

EFEL7002 was isolated from Korean *meju* (fermented soybean) and identified as *T. halophilus,* with 99.85% similarity. The strain is safe for use in food as it is a non-hemolytic and non-biogenic amine producer. EFEL7002 is tolerant to gastrointestinal conditions and can adhere to Caco-2 cells. This strain showed antioxidant, anti-inflammatory, and protective effects against the human gut epithelial barrier. EFEL7002 grew well in media containing 0–18% NaCl showing maximum cell densities in 6% or 12% NaCl.

**Conclusions:**

*T. halophilus* EFEL7002 can be used as a health-promoting probiotic starter culture for various salty fermented foods.

**Supplementary Information:**

The online version contains supplementary material available at 10.1186/s12866-022-02561-7.

## Background

*Tetragenococcus halophilus* is a gram-positive, catalase-negative, and oxidase-negative halophilic lactic acid bacterium (LAB) that is distinguished from bacterial species because it can survive under high salt and pH conditions [1]. It has been isolated from salty fermented foods, such as soy sauce [2], soybean paste [3], jeotgal [4], and fish sauce [5] and is reported to produce organic acids at NaCl concentrations ranging from 5 to 20% [6]. *T. halophilus* contributes to the umami taste during soy sauce fermentation by producing aspartic acid, glutamic acid, dipeptide N-succinyl-glutamic acid, and alanine [2]. Besides, it produces volatile compounds, such as benzeneacetaldehyde and 2-methyl-propanal, which provide a floral scent [7, 8]. Owing to these characteristics, *T. halophilus* is regarded as a suitable starter to improve the flavor profile of high-salt fermented foods.

Probiotics are living microorganisms that, when consumed in appropriate amounts, exert beneficial effects on the host [9]. Some beneficial effects of probiotic consumption include prevention of intestinal infections by pathogenic bacteria [10], immune regulation [11], anticancer activity [12], and detoxification [13]. Various LAB, including *Lactobacillus* spp., are probiotic [14] and are used as starter cultures for various fermented foods, including yogurt [15] and cheese [16]. Since several fermented foods are mainly produced using lactic acid bacteria and often consumed with daily meals, they are regarded as the best matrices to deliver probiotics [17, 18]. However, because the growth of most LAB is delayed under high-salt conditions [19], their use as probiotic starter cultures for high-salt fermented foods is limited. In this context, *T. halophilus* is considered as a potential candidate probiotic LAB suitable for the fermentation of high-salt foods. Therefore, it is necessary to develop a probiotic *T. halophilus* that can be used not only as a starter in high-salt fermented foods, but also as a microorganism to confer health benefits to human.

Biogenic amines (BAs) are produced in fermented foods by removal of the alpha carboxyl group from amino acids by decarboxylases of microorganisms [20]. However, these BAs can cause neurotoxicity when ingested in large amounts, with symptoms such as nausea, headaches, rashes, and increased, and decreased blood pressure, leading to safety issues [21]. LAB are major producers of BAs, and it has been reported that BAs may play a role in acid stress resistance by increasing the consumption of hydrogen ions under low pH conditions [22]. BAs, such as tyramine and histamine, were detected in large amounts in protein-rich fermented foods [23] and 45 strains of *T. halophilus* isolated from naturally fermented doenjang (soybean paste) produced the BAs cadaverine, putrescine, and tyramine [24]. Therefore, the use of a *T. halophilus* strain lacking BA-producing ability as a starter culture is suggested to prevent BA accumulation in fermented foods [4].

Here, we report the probiotic effects of a BA-nonproducing *T. halophilus* strain EFEL7002 under high-salt fermentation conditions. Initially, safety factors (hemolytic activity and BA-producing ability) and gastrointestinal stability (resistance to acid and bile salt, and epithelial cell adhesion capacity) were evaluated. Second, health-promoting functions, such as epithelial cell protection and antioxidant and anti-inflammatory activities, were analyzed. Finally, the biochemical characteristics and fermentation profiles of the EFEL7002 strain under high-salt conditions were analyzed.

## Results

### Isolation and phylogenetic analysis of EFEL7002

**Fig. 1:**
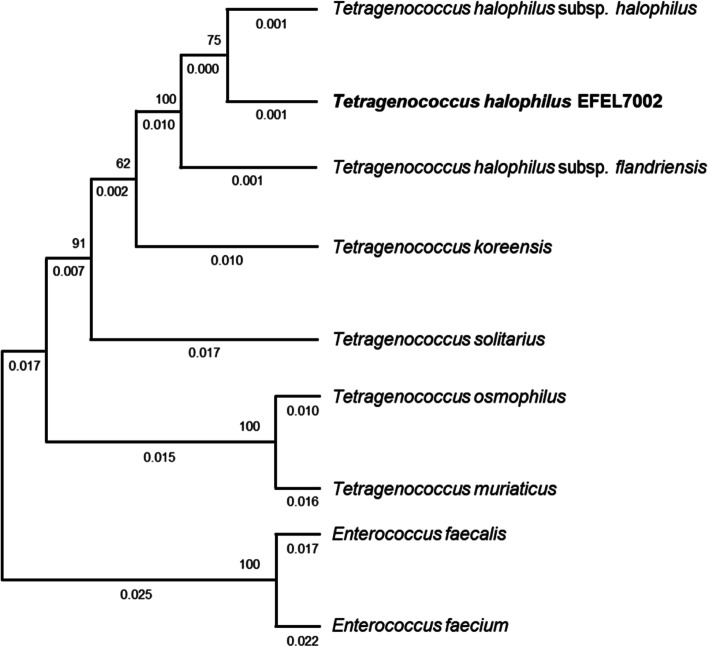
Phylogenetic tree of *Tetragenococcus halophilus* EFEL7002 compared to reference strains based on 16S rRNA sequences

*T. halophilus* EFEL7002 was isolated from traditional Korean *meju*, which serves as a preculture of several Korean condiments, such as *doenjang* (soybean paste), *ganjang* (soy sauce), and *gochujang* (chili paste). Of the 17 different *T. halophilus* isolates obtained from *meju*, EFEL7002 was selected after comparison of BA productivity, stability under gastrointestinal conditions, and inhibitory activity against nitric oxide (NO) production. Phylogenetic analysis based on 16S rRNA gene sequences showed that the strain EFEL7002 formed a tight phyletic lineage with the type strains of *T. halophilus* subsp. *halophilus* (Fig. 1), showing high similarity (99.85%). This indicates that the EFEL7002 strain isolated from *meju* is a member of the species *T. halophilus*.

### FEL7002, a non-hemolytic and non-BA producing strain, is safe for use in food fermentation

**Fig. 2:**
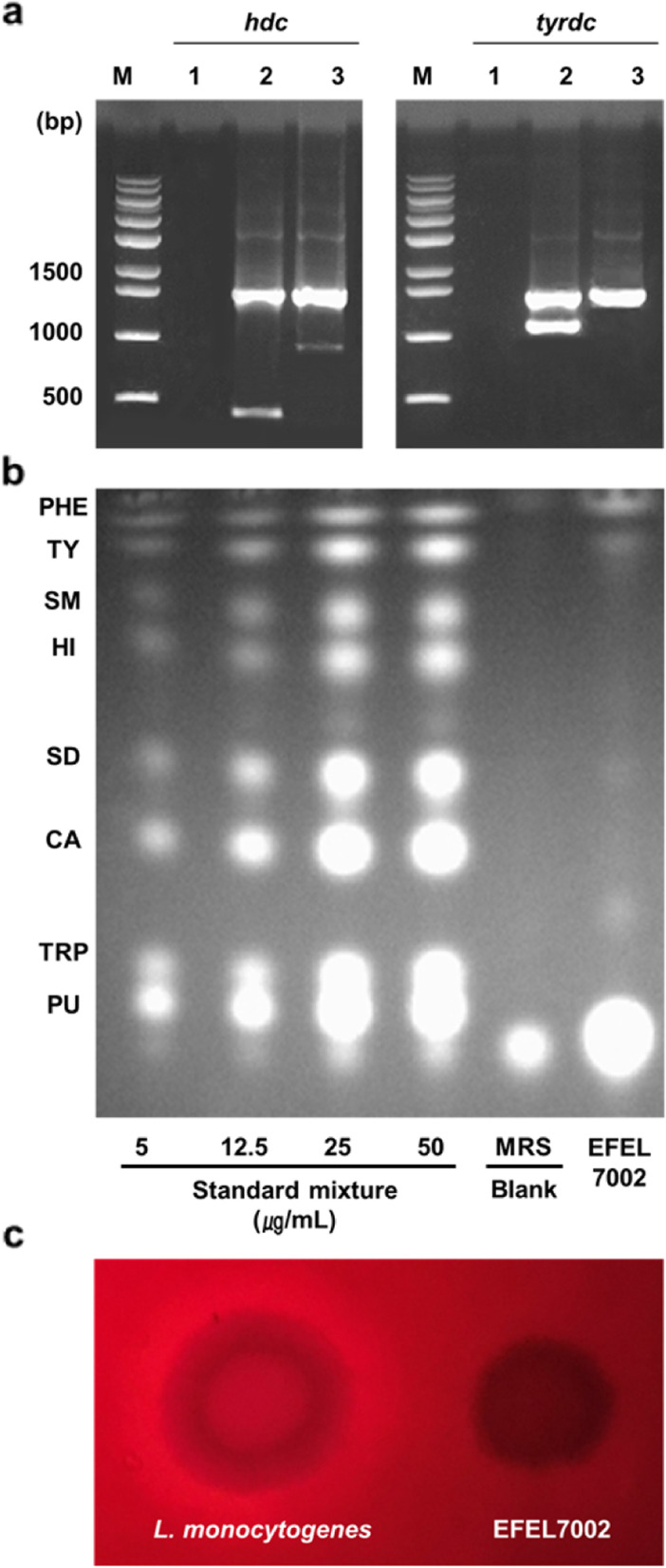
Safety assessment for *Tetragenococcus halophilus* EFEL7002. **a** Detection of genes related to biogenic amine production. Lane M, 1 kb DNA marker; lane 1, negative control with no template DNA; lane 2, positive control for the genes *hdc* (encoding histidine decarboxylase, 440 bp), and *tyrdc* (encoding tyrosine decarboxylase, 1100 bp) from *Limosilactobacillus reuteri* ATCC 23272 and *Enterococcus faecalis* KCCM 11729, respectively; and lane 3, EFEL7002. The 16S rRNA gene (1530 bp) was also amplified. The gel image was cropped because the gel contained samples that are not discussed in this study. **b** Thin layer chromatography detection of biogenic amines produced by *T. halophilus* EFEL7002. Biogenic amine standards of 5, 12.5, 25, and 50 μg/mL were prepared for the following: phenylethylamine, tyramine, spermine, histamine, spermidine, cadaverine, tryptamine, and putrescine. **c** Hemolytic activity of *T. halophilus* EFEL7002 was measured in brain heart infusion broth supplemented with 7% horse blood. Right, EFEL7002 strain; left, the positive control, *Listeria monocytogenes*, showing a clear zone around the cell drop. Abbreviations: PHE, phenylethylamine; TY, tyramine; SM, spermine; HI, histamine; SD, spermidine; CA, cadaverine; TRP, tryptamine; and PU, putrescine

To evaluate the safety of the EFEL7002 strain, BA production capacity and hemolytic activity were assessed. First, decarboxylase genes (*hdc* and tyrdc) related to BA synthesis were analyzed. As shown in Fig. 2a, the EFEL7002 strain did not show bands for *hdc* and *tyrdc,* genes related to production of histamine and tyramine, respectively, in agarose gel electrophoresis. However, bands for *hdc* and *tyrdc* were detected in the positive control strains *Limosilactobacillus reuteri* ATCC 23272 and *Enterococcus faecalis* KCCM 11729, respectively. Second, when the cell culture supernatant of EFEL7002 was separated using thin layer chromatography (TLC), no corresponding spots for BAs were detected, while eight standard compounds showed distinct spots on TLC in a concentration-dependent manner (Fig. 2b). Third, when live cells of EFEL7002 were inoculated on blood agar plates to test hemolytic activity, the strain did not show any clear zones around the colonies, whereas *Listeria monocytogenes* showed a clear zone of hemolytic activity (Fig. 2c). These results indicate that the EFEL7002 strain is safe for use in food fermentation.

### EFEL7002 exhibits acid and bile salt tolerance

**Fig. 3:**
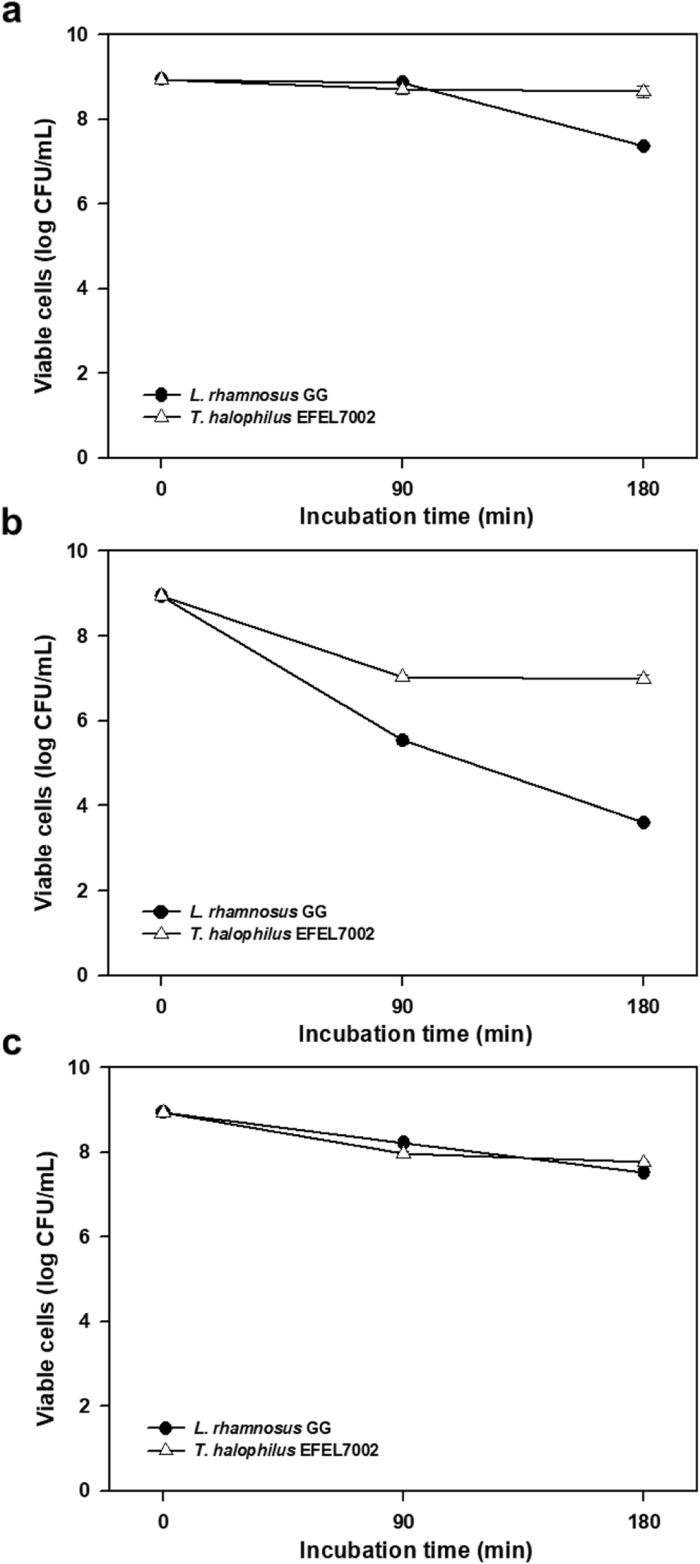
Viability of *Tetragenococcus halophilus* EFEL7002 under various simulated gastrointestinal conditions. The conditions tested include: (**a**) pH 3.0, (**b**) pH 2.5, and (c) 0.3% bile salt concentration. Results are expressed as the mean ± standard deviation (*n* = 3). Significant differences compared with *Lacticaseibacillus rhamnosus* GG are represented with asterisks (^*^p < 0.05, ^***^p < 0.001)

To test the stability of EFEL7002 under gastrointestinal conditions, its tolerance to acid and bile salts was measured. As shown in Fig. 3a and b, the EFEL7002 strain exhibited considerably higher resistance at pH 3.0 (8.6 log CFU/mL) and pH 2.5 (6.9 log CFU/ mL) after 180 min incubation than *Lacticaseibacillus* rhamnosus GG (LGG), which lost viability at both pH 3 (6.8 log CFU/mL) and pH 2.5 (3.3 log CFU/mL). Similarly, when bile tolerance was measured in 0.3% bile salt solution, cell viability of EFEL7002 was substantially higher (7.8 log CFU/mL) after 180 min incubation compared to LGG (7.0 log CFU/mL) (Fig. 3c). These results indicate that EFEL7002 can survive under human gastrointestinal conditions; therefore, most of the ingested cells of this strain can reach the large intestine in viable forms.

### EFEL7002 adheres to intestinal epithelial cells

**Fig. 4:**
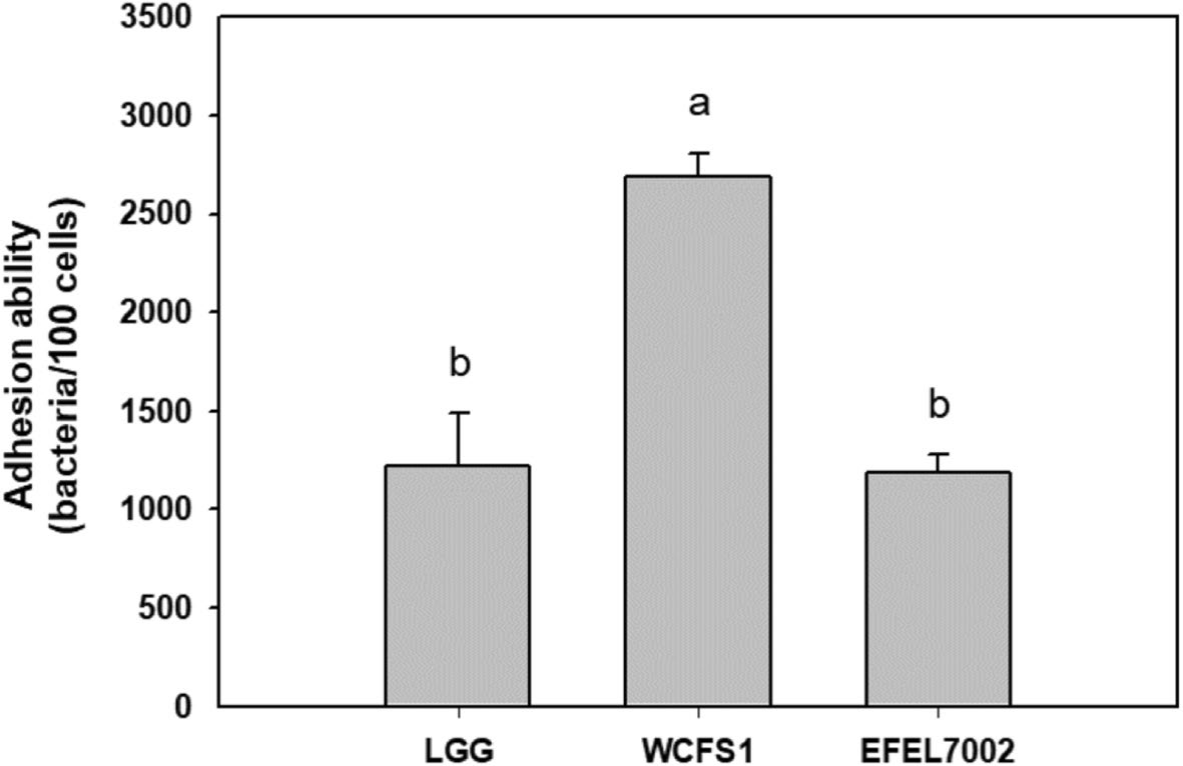
Intestinal adhesion capacity of *Tetragenococcus halophilus* EFEL7002. The colonic epithelial cell line Caco-2 was used to assess intestinal adhesion capacity. Results are expressed as the numbers of adhered bacteria per 100 Caco-2 cells (*n* = 3). *Lacticaseibacillus* rhamnosus GG and *Limosilactobacillus plantarum* WCFS1 were used as the positive controls. Different letters on the error bars indicate significant differences (*p* < 0.05). Abbreviations: LGG, *Lacticaseibacillus* rhamnosus GG; WCFS1, *Limosilactobacillus plantarum* WCFS1

To evaluate the ability of EFEL7002 to adhere to intestinal epithelial cells, the strain was incubated on a Caco-2 cell monolayer. As shown in Figs. 4, and 1,187 ± 88 EFEL7002 cells adhered to 100 Caco-2 cells. The adhesion capacity of EFEL7002 was similar to that of LGG, whereas the adhesion capacity of *Lactiplantibacillus plantarum* WCFS1 (WCFS1) was higher than that of LGG. This demonstrates that EFEL7002 has an adhesion capacity comparable to that of a commercial probiotic strain.

### EFEL7002 exerts protective effects against H_2_O_2_-induced intestinal permeability

**Fig. 5:**
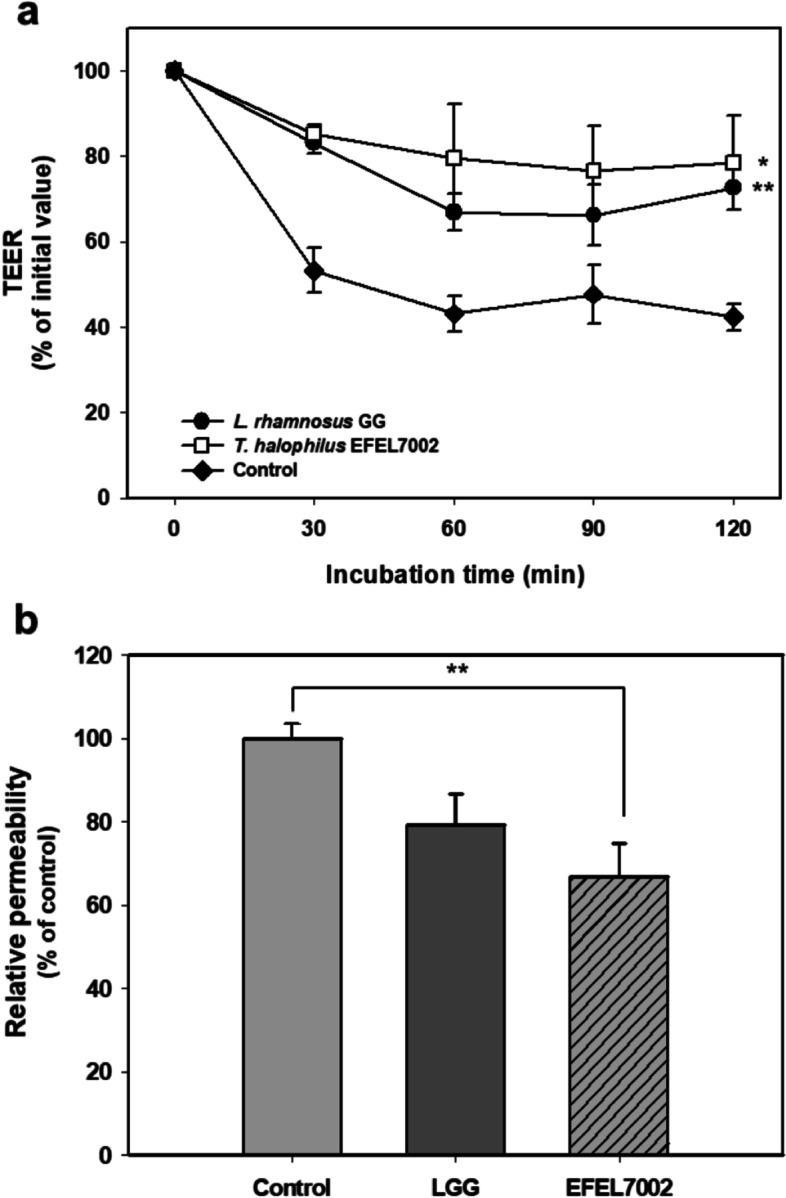
Protective effects of *Tetragenococcus halophilus* EFEL7002 against H_2_O_2_-induced permeabilization of Caco-2 cell monolayers. Caco-2 cell monolayers were pretreated with bacterial cells for 30 min and were then exposed to 100 μM H_2_O_2_. **a** Transepithelial electrical resistance was measured every 30 min until 2 h after H_2_O_2_ treatment. **b** FITC-dextran fluorescence intensity was measured after 4 h and expressed in % compared to the control value. *Lacticaseibacillus rhamnosus* GG (LGG) was used as the positive control. ∗*p* < 0.05, ∗∗*p* < 0.01 compared with the control. Abbreviations: LGG, *Lacticaseibacillus* rhamnosus GG; TEER, Transepithelial electrical resistance

To assess the protective activities of EFEL7002 on damaged epithelial cell monolayers, EFEL7002 cells were incubated with hydrogen peroxide (H_2_O_2_)–treated confluent Caco-2 cells, and the transepithelial electrical resistance (TEER) value and fluorescein isothiocyanate–dextran (FITC-dextran) flux were measured (Fig. 5). EFEL7002 strain showed a substantial protective effect against H_2_O_2_-induced epithelial damage (Fig. 5a); the addition of H_2_O_2_ to Caco-2 cell monolayers caused a decrease in TEER (42.34%) after 120 min incubation, but EFEL7002 was better at preventing this decrease (> 78.5%) compared to LGG (72.6%). Similar results were also observed when the paracellular permeability of Caco-2 cell monolayers was measured using fluorescence levels of FITC-dextran passing through Caco-2 cells; EFEL7002 showed lower fluorescence levels (66.9%) than LGG (79.2%) and the control (100%, *p* < 0.01) (Fig. 5b). These results clearly show that EFEL7002 exerts a protective effect on Caco-2 cell monolayers against H_2_O_2_-induced oxidative damage.

### EFEL7002 possesses antioxidant activity

**Fig. 6:**
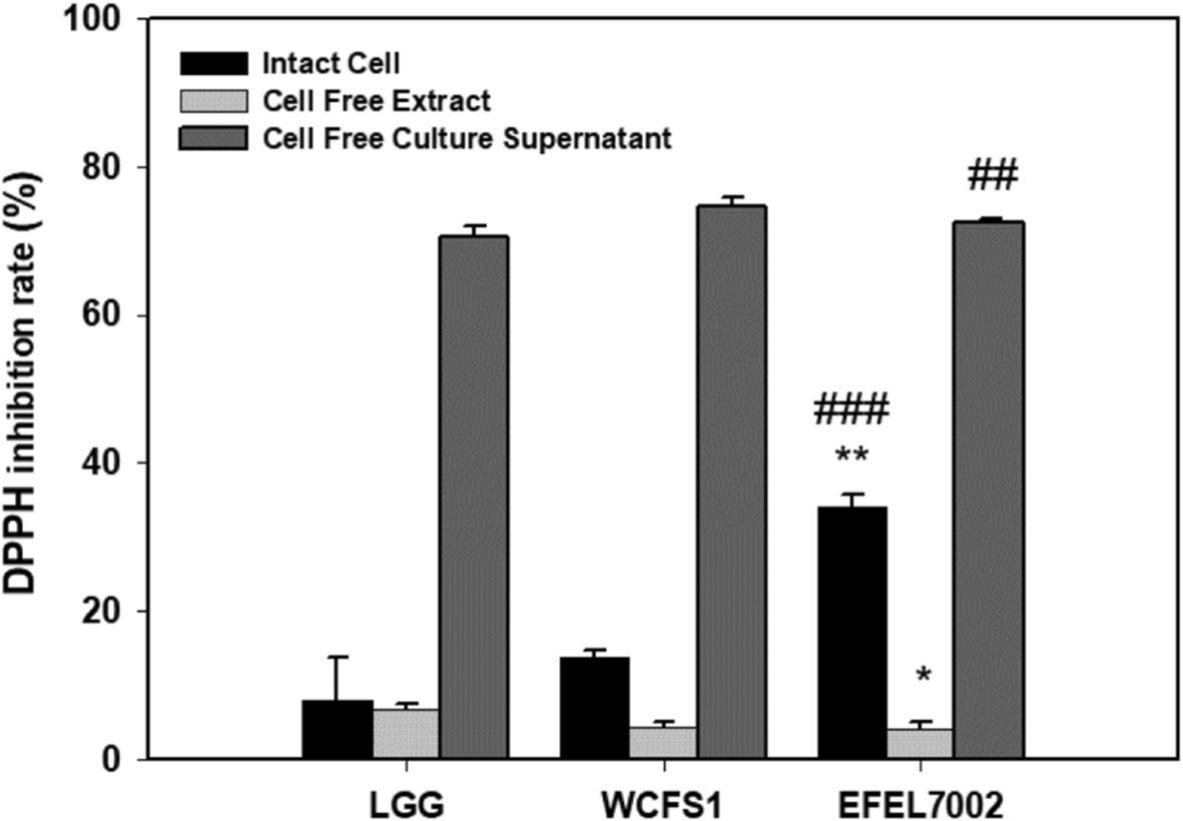
Antioxidant activity of *Tetragenococcus halophilus* EFEL7002 measured by the DPPH inhibition assay. Fractions of intact cells, cell-free extract, and cell-free culture supernatant were used. Significant differences compared with *Lacticaseibacillus rhamnosus* GG are represented using asterisks (^*^p < 0.05, ^**^p < 0.01) or with *Limosilactobacillus plantarum* WCFS1 are represented using hashtags (^##^*p* < 0.01, ^###^*p* < 0.001). Abbreviations: DPPH, 2,2-diphenyl-1-picrylhydrazyl; LGG, *Lacticaseibacillus* rhamnosus GG; WCFS1, *Limosilactobacillus plantarum* WCFS1

Following the above analysis, we further evaluated the antioxidant activity of EFEL7002 by measuring 2,2-diphenyl-1-picrylhydrazyl (DPPH) scavenging activity using intact cells, cell-free extracts, and cell-free culture supernatants (Fig. 6). All three fractions showed DPPH scavenging activity with the highest observed in cell-free culture supernatant (72.3%), followed by intact cells (34.0%) and cell-free extract (3.9%). Compared with the controls, the intact cells of EFEL7002 exhibited considerably higher activity (34.0%) than that of LGG (7.9%) and WCFS1 (13.7%), suggesting the presence of antioxidative factors in the cellular structure of EFEL7002. This suggests that both intact cells and cell-free culture supernatant of EFEL7002 can exert protective effects against oxidative stress in the human intestine.

### EFEL7002 exerts inhibitory effects against NO production

**Fig. 7:**
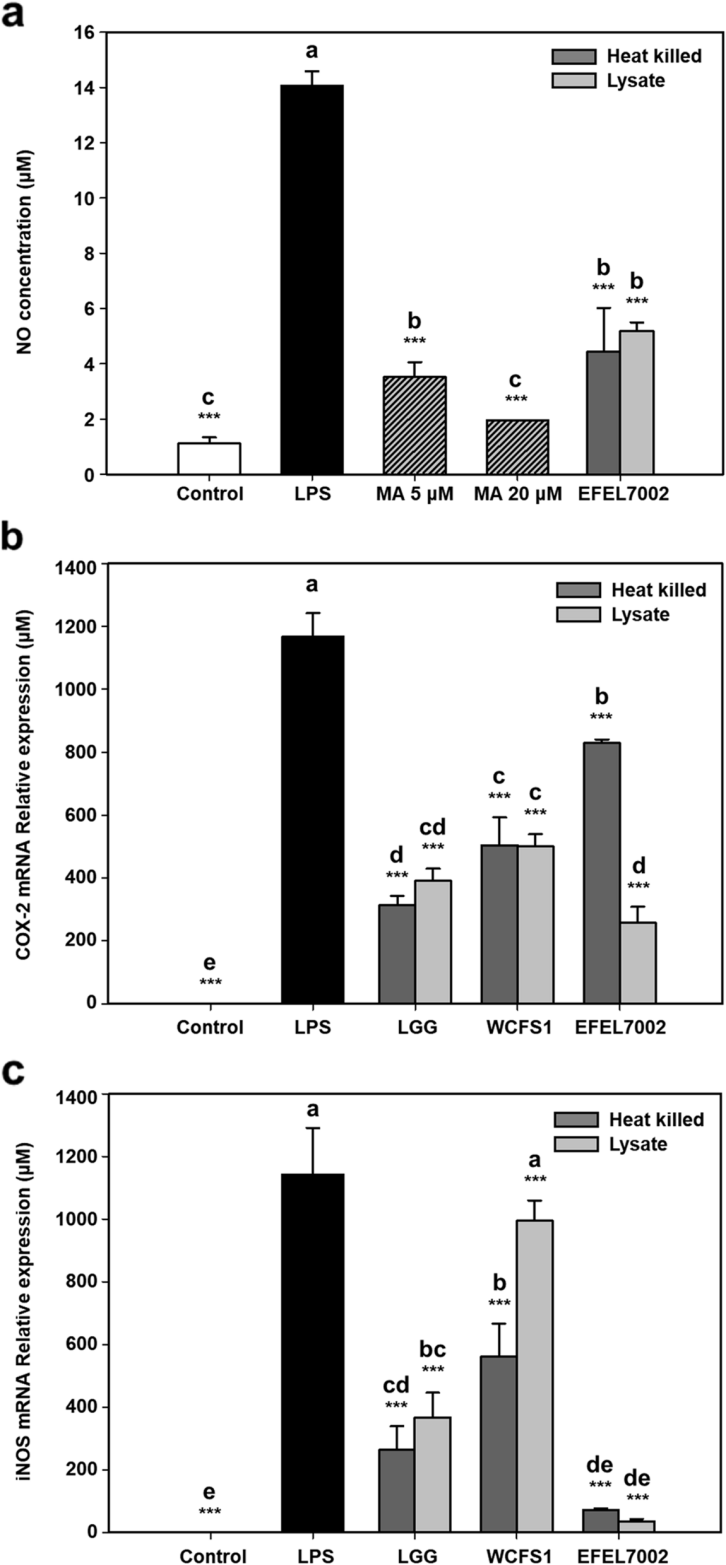
Anti-inflammatory activity of *Tetragenococcus halophilus* EFEL7002 in LPS-induced RAW 264.7 cells. Effect of heat-killed cells and lysates of *T. halophilus* EFEL7002 on (**a**) nitric oxide production (**a**), (b) mRNA expression of COX-2, and (**c**) mRNA expression of iNOS. Different letters on the error bars indicate significant differences (*p* < 0.05) according to Duncan’s multiple range test. In addition, significant differences with the LPS positive group are represented using asterisks (***p < 0.001). Abbreviations: LGG, *Lacticaseibacillus* rhamnosus GG; LPS, lipopolysaccharide; MA, methyl arginine; NO, nitric oxide; WCFS1, *Limosilactobacillus plantarum* WCFS1

To evaluate the anti-inflammatory effects of EFEL7002, the inhibitory activities of heat-killed cells and cell lysate against NO production were analyzed in lipopolysaccharide (LPS)-induced RAW 264.7 cells (Fig. 7a). Both heat-killed cells and lysates of EFEL7002 considerably inhibited NO production (4.1 and 5.1 μM, respectively), comparable to the inhibition observed with 5 μM methyl arginine, an NO synthase inhibitor. In addition, we performed quantitative real time polymerase chain reaction (RT-qPCR) to investigate whether the inhibitory ability of EFEL7002 on NO production was associated with the transcript levels of *COX-2* (Fig. 7b) and *iNOS* (Fig. 7c) in RAW 264.7 cells. Heat-killed cells and lysates of EFEL7002 considerably inhibited the mRNA expression levels of both *COX-2* (256.9 and 51.9 μM, respectively) and *iNOS* (72.3 and 35.7 μM, respectively) (*p* < 0.05). In particular, iNOS inhibition by EFEL7002 was significantly higher than that of LGG and WCFS1 (p < 0.05). These results show that treatment with heat-killed cells and lysates of EFEL7002 inhibit NO production by repressing the mRNA expression of *COX-2* and *iNOS* in RAW 264.7 cells.

### EFEL7002 induces expression of cytokines

**Fig. 8:**
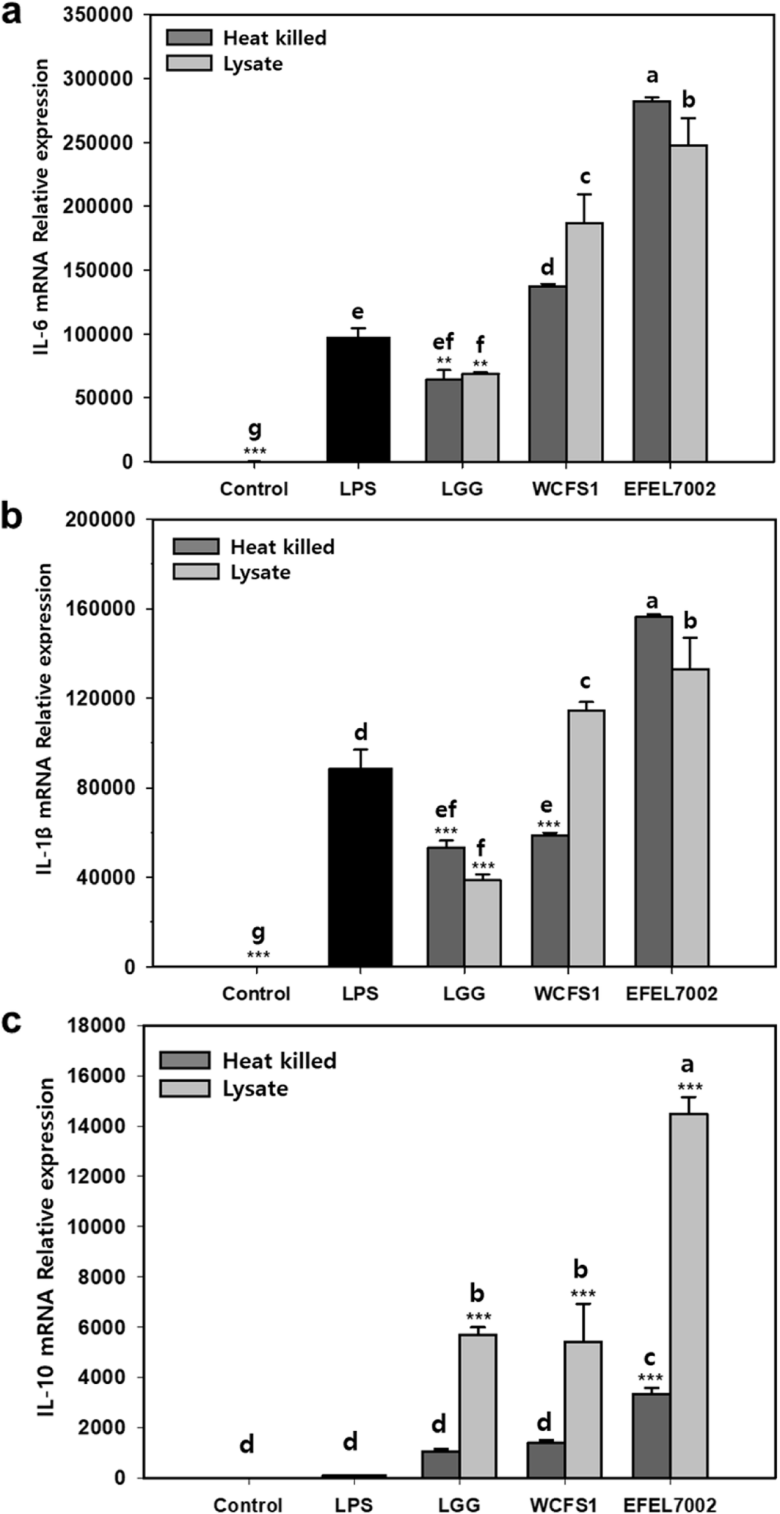
Expression of cytokine genes in *Tetragenococcus halophilus* EFEL7002: (**a**) IL-6, (**b**) IL-1β, (c) IL-10. Different letters on the error bars indicate significant differences (*p* < 0.05) according to Duncan’s multiple range test. In addition, significant differences are present with LPS positive group (**p < 0.01, ***p < 0.001). Abbreviations: LGG, *Lacticaseibacillus* rhamnosus GG; LPS, lipopolysaccharide; WCFS1, *Limosilactobacillus plantarum* WCFS1

To further analyze the anti-inflammatory activity of EFEL7002, the mRNA expression levels of pro-inflammatory cytokines, interleukin (IL)-6 and IL-1β, and anti-inflammatory cytokine IL-10, in LPS-induced RAW 264.7, were measured by RT-qPCR (Fig. 8). Treatment with both heat-killed cells and lysates of EFEL7002 upregulated the mRNA expression of IL-6 and IL-1β (Fig. 8a and b), and IL-10 (Fig. 8c). In particular, lysates of EFEL7002 substantially induced the expression of IL-10 compared to the positive controls (LGG and WCFS1). These results suggest that the induction of IL-10 by EFEL7002 may be associated with the inhibition of NO production, consequently exerting anti-inflammatory effects on immune cells.

### Microbial and biochemical characteristics of EFEL7002

**Fig. 9:**
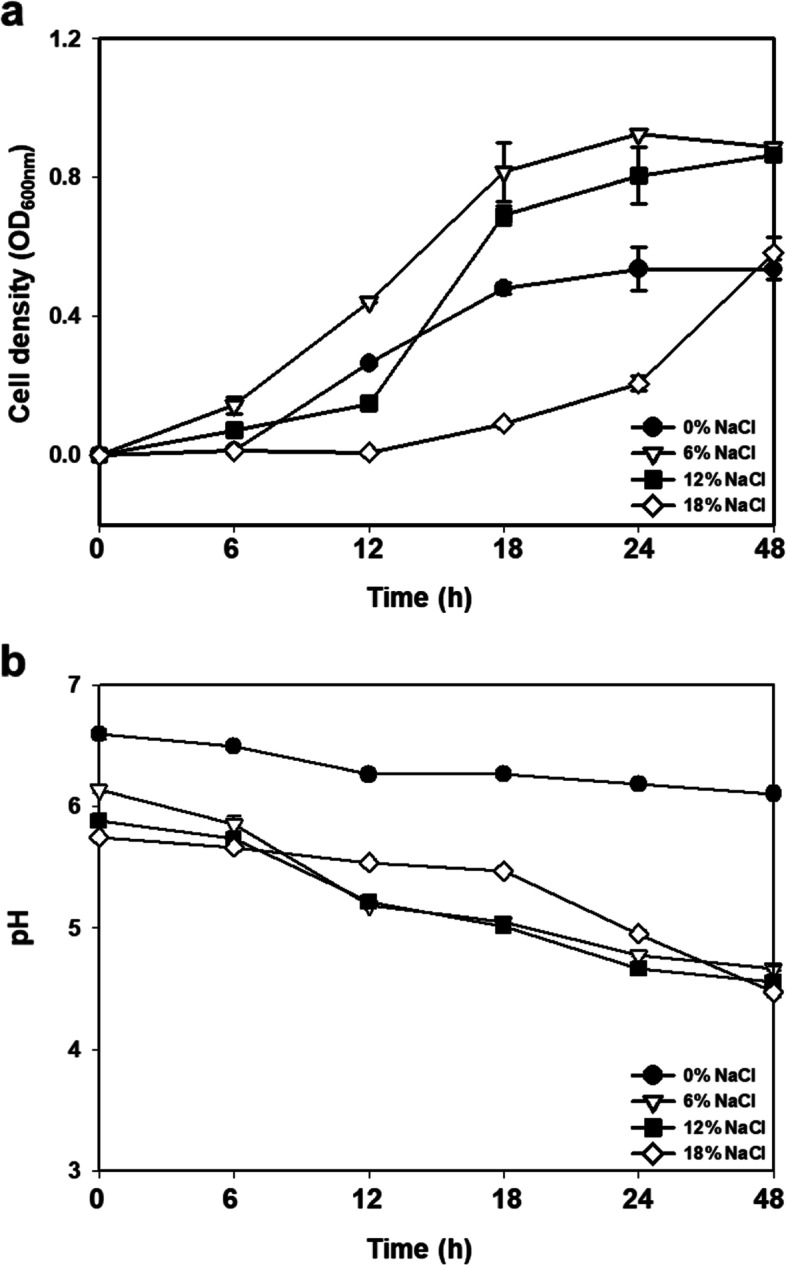
Growth rate (**a**) and pH change (**b**) of *Tetragenococcus halophilus* EFEL7002 at different NaCl concentrations

**Fig. 10:**
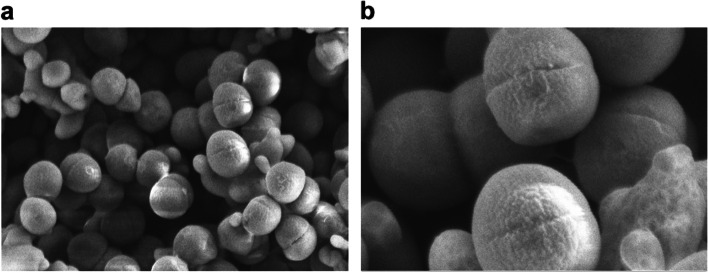
Scanning electron microscope images of *Tetragenococcus halophilus* EFEL7002. (**a**) 50,000×. (**b**) 150,000×

In addition to the probiotic traits of EFEL7002, the general microbial and biochemical characteristics of the strain were analyzed. As shown in Fig. 9, EFEL7002 was able to grow in different salt concentrations (0–18% NaCl) and decreased the pH from pH 6 to pH 4.6 (except in 0% NaCl) by synthesizing lactic acid (data not shown). Notably, EFEL7002 showed maximum cell densities in media containing 6 and 12% NaCl, demonstrating that *T. halophilus* EFEL7002 is a true halophilic LAB that is suitable for fermentation of high-salt foods. Additionally, when the morphological characteristics of EFEL7002 were inspected using a scanning electron microscope (Fig. 10), the cells had a typical non-motile cocci form with a size of 0.8–1 μm and showed a division ring called the Z-ring, which is made of a filamentous tubulin analog, at the mid cell. Finally, the biochemical characteristics of EFEL7002 were investigated by analyzing its sugar utilization ability (Table 1). Among the 49 carbohydrates tested, EFEL7002 metabolized 20 different carbohydrates. EFEL7002 could metabolize glucopyranoside, melibiose, sucrose, and raffinose, which the type strain *T. halophilus* T11 could not. This finding highlights that *T. halophilus* EFEL7002, isolated from a Korean soy *meju*, is a novel strain with biochemical characteristics different from those of the type strain. *T. halophilus* EFEL7002 was deposited in the Korean Agricultural Culture Collection (KACC, Wanju, South Korea) under the accession number KACC 81138BP.

**Table 1:** Carbohydrate utilization pattern of *Tetragenococcus halophilus* EFEL7002 and T11^Ta^

Carbohydrate	EFEL7002	T11^T^
Glucopyranoside	+^b^	–
Melibiose	+	–
Sucrose	+	–
Trehalose	–	+
Raffinose	+	–

^a^All strains were positive for L-arabionose, D-ribose, D-galactose, glucose, fructose, mannose, N-acetyl-glucosamine, amygdalin, arbutin, esculin, salicin, cellobiose, maltose, gentiobiose, turanose and tagatose. All strains were negative for glycerol, erythritol, D-arabinose, D-xylose, L-xylose, D-adonitol, D-xylopytanoside, sorbose, rhamnose, dulcitol, inositol, manntiol, sorbitol, mannopyranoside, lactose, inulin, melezitose, starch, glycogen, xylitol, lyxose, D-fucose, L-fucose, D-arabitol, L-arabitol, gluconate, 2-ketogluconate and 5-ketogluconate (API 50CH)

b+, Positive; −, negative

## Discussion

Adequate amounts of salt inhibit the growth of harmful bacteria in fermented foods and contribute to the flavor, texture, and moisture content [25]. Cheese is a representative fermented dairy product and pickled cheeses, such as domiati and feta contain 4–6% salt [26]. Soy sauce [27], doenjang [28], broad bean paste [29], and sweet bean paste [30] are representative fermented legume products with added salt in the range of 0.3–26%. Although LAB, such as Lactobacilli, Lactococci, *Leuconostoc* spp., *Weissella* spp., and *Oenococcus* spp., are widely used as starters in food fermentation, they are not generally used for high-salt foods due to their delayed growth rates [19]. However, *T. halophilus* is a halophilic lactic acid bacterium that is resistant to salt stress by defense mechanisms such as the increase of unsaturated fatty acids in the cell membrane, the accumulation of specific substances like citrulline, and the regulation of signaling molecules [31]. Among LABs from traditional Chinese fermented soybean paste, most of the halophilic LAB were identified as *T. halophilus* [32]. Even though *Staphylococcus* spp. can survive a wide range of salt concentrations, *Tetragenococcus* spp. have been reported to have a higher optimum salt concentration [33]. The present study provides evidence that *T. halophilus* EFEL7002 is a suitable starter for high-salt foods because it is safe and can grow in high NaCl concentrations (6–18%) (Fig. 9). In particular, EFEL7002 can utilize raffinose (Table 1), which is present in legumes or vegetables.

The development of probiotic starters is an emerging theme among scientists and industries because not only do they improve the quality of fermented foods by contributing to the flavor profile and texture, but also exert health-promoting effects [17, 18]. In the present study, we isolated a potential probiotic starter for protein-rich foods as soybean is considered an ideal food matrix for delivering probiotics in daily meals.

The primary requirement for the development of a starter culture for food fermentation is safety. In this regard, we analyzed hemolytic activity and BA production in *T. halophilus* EFEL7002. In a previous study, hemolytic activity and BA-producing ability were reported for LAB isolated from naturally fermented foods [24, 34]. Hemolysin, which lyses red blood cells, is a pore-forming toxin used by major pathogens and contributes greatly to in vivo toxicity [35, 36]. BAs are low-molecular-weight nitrogenous organic bases that can accumulate in high concentrations in food and exert harmful effects on the human body, such as scombroid poisoning [21, 37]. The present study showed that *T. halophilus* EFEL7002 does not harbor any BA-producing genes (*hdc* and *tyrdc*) and does not produce BA products, from eight tested compounds (Fig. 2). In addition, the EFEL7002 strain showed no hemolytic activity, indicating its safety as a starter for food fermentation.

Another requirement for probiotics is gastrointestinal stability or epithelial cell adhesion capacity after ingestion. Our results showed that EFEL7002 has high gastrointestinal stability and epithelial cell adhesion capacity. EFEL7002 exhibited higher survival rates (6.9 and 7.8 log CFU/mL) in gastrointestinal conditions (pH 2.5 and 0.3% bile acid, respectively) after 180 min when compared to LGG (3.3 and 7.0 log CFU/mL, respectively) (Fig. 3). Additionally, 1187 EFEL7002 cells adhered to 100 Caco-2 cells, exhibiting an epithelial cell adhesion capacity similar to that of LGG (Fig. 4). These results indicated that EFEL7002 could colonize and function in the human digestive tract at a level similar to that of commercial probiotics.

Furthermore, our results showed that *T. halophilus* EFEL7002 exerts health-promoting effects. The intestinal epithelial cell membrane is known to be important for its functional and physical barrier roles, as the influx of harmful molecules caused by increased paracellular permeability under various pathological conditions can lead to diseases, such as type I diabetes and systemic lupus erythematosus [38]. In the present study, the protective effect of EFEL7002 on barrier integrity was validated using an epithelial cell permeation model. EFEL7002 maintained low extracellular permeability against H_2_O_2_ stress, demonstrating barrier-protective function (Fig. 5). Meanwhile, various harmful factors, such as pathogens that constantly interact with intestinal epithelial cells in the gastrointestinal tract, can induce free radical production and trigger oxidative stress and inflammatory responses [39]. In the present study, intact EFEL7002 cells had significantly higher antioxidant activity than LGG and WCFS1, suggesting that the cell membrane component of EFEL7002 cells has superior antioxidant activity. In a previous study [40], Lactobacilli were able to survive for more than 8 h in media containing H_2_O_2_, which causes oxidative damage, and intact cells had higher DPPH radical scavenging activity than LGG, which is consistent with our results. In addition, our results showed that EFEL7002 had anti-inflammatory activity. Inflammatory bowel disease (IBD) is a chronic disease characterized by deteriorating inflammation of the gastrointestinal tract, including Crohn’s disease and ulcerative colitis [41]. IBD pathogenesis is associated with host innate immunity, adaptive immunity, intestinal microbiota, and leaky gut function [42]. Our results showed that both heat-killed cells and lysates of EFEL7002 suppressed NO production in LPS-induced RAW 264.7 cells (Fig. 7). NO is a cellular signaling molecule associated with insulin secretion and intestinal peristalsis; however, excessive NO production can lead to hypertension, stroke, and inflammatory responses [43]. NO is produced by NO synthase (NOS), which includes nNOS, eNOS, and iNOS. In particular, iNOS produces a large amount of NO, is regulated by cytokines, and is associated with tumor regulation, such as malignant transformation and angiogenesis [44]. Therefore, the ability of EFEL7002 to inhibit NO production (Fig. 7) could be related to suppression of iNOS mRNA transcription by EFEL7002 (Fig. 8). In addition, EFEL7002 decreased Cox-2 mRNA expression. COX-2 is regulated by pro-inflammatory factors, and its mRNA expression has been reported to be associated with pro-inflammatory prostaglandins, edema, and hyperalgesia responses [45]. Moreover, we analyzed the anti-inflammatory activity of EFEL7002 at the level of cytokine induction. Raw 264.7 cells treated with LPS alone significantly induced pro-inflammatory cytokines, IL-6 and IL-1β, but did not induce the anti-inflammatory cytokine IL-10. In contrast, heat-killed cells and lysates of EFEL7002 were able to strongly induce IL-10, suggesting an anti-inflammatory effect. IL-10 is a pleiotropic cytokine that inhibits the functions of T cells, monocytes, and macrophages and is known to decrease and inhibit inflammatory responses [46]. IL-10 alleviated LPS-induced fetal death in 3 month pregnant rats [47] and growth retardation and chronic enteritis were reported in IL-10-deficient mice [48].

## Conclusions

In the present study, we report that *T. halophilus* EFEL7002 is a halophilic bacterium that has valuable probiotic activities, such as intestinal epithelial cell protective effects, antioxidant and anti-inflammatory activities. Therefore, *T. halophilus* EFEL7002 can be used as a health-promoting probiotic starter culture for various salty fermented foods. To improve the quality of high-salt fermented food, further studies of fermentation properties and metabolites synthesis are needed when applied as starter.

## Methods

### Isolation and phylogenetic analysis of strain EFEL7002

For isolation of the strain, Korean traditional *meju* samples were serially diluted in sterile 0.85% NaCl solution, spread on bromophenol blue-modified de Man-Rogosa Sharpe (MRS; BD Difco™, Sparks, MD, USA) agar medium containing 0.1 μg/mL cycloheximide, and cultured anaerobically at 37 °C. To identify the isolate, PCR amplification of the 16S rRNA gene was performed using the universal primers 27F (5-AGAGTTTGATCMTGGCTCAG-3) and 1492R (5-GGTTACCTTGTTACGACTT-3). To construct phylogenetic tree, Molecular Evolutionary Genetics Analysis was used across Computing Platforms (Mega X; https://www.megasoftware; Accessed 9 Sep, 2021) with 16S rRNA sequences of *T. halophilus* spp. and *Enterococcus* spp. obtained from EzBioCloud database (https://www.ezbiocloud.net; Accessed 9 Sep, 2021).

### Microorganisms and culture conditions

*T. halophilus* EFEL7002 was cultured in MRS broth containing 6% (w/v) NaCl at 37 °C. LGG, WCFS1, and *L. reuteri* ATCC 23272 were cultured in MRS broth at 37 °C. *E. faecalis* KCCM 11729 and *L. monocytogenes* KCTC 3569 were cultured in brain heart infusion (BHI; BD Difco™) broth at 37 °C. All strains were stored in 15% (v/v) glycerol solution at − 80 °*C. prior* to the experiments, each strain was cultured in the optimal culture medium under optimal conditions for 24 h.

### Detection of BA related genes

To assess the safety of *T. halophilus* EFEL7002, genes related to BA production and the 16S rRNA gene in genomic DNA were detected using multiplex PCR. DNA was extracted from the bacteria using the Genomic DNA extraction kit (SolGent, Daejeon, Korea). The *hdc* (encoding histidine decarboxylase) and *tyrdc* (encoding tyrosine decarboxylase) genes were amplified using the following primer pairs: *hdc*: HDC3 (5-GATGGTATTGTTTCKTATGA-3) and HDC4 (5-CAAACACCAGCATCTTC-3); *tyrdc*: TD2 (5-ACATAGTCAACCATRTTGAA-3) and TD5 (5-CAAATGGAAG AAGAAGTAGG-3). 16S rRNA gene was amplified using the universal primers 27F and 1492R. In multiplex PCR, each BA gene was amplified at the same time as the 16S rRNA gene in a PCR tube by adding each of the corresponding primer set. The genomic DNA of EFEL7002 was amplified using the 16S rRNA gene as a positive control for the PCR reaction. The PCR reaction conditions were as follows: initial denaturation at 95 °C for 5 min; followed by 32 cycles of denaturation at 95 °C for 45 s, annealing at 58 °C for 45 s, and extension at 72 °C for 75 s; and final extension at 72 °C for 5 min. The genomic DNA of *L. reuteri* ATCC 23272 and *E. faecalis* KCCM 11729 were used as positive controls for *hdc* and *tyrdc*, respectively.

### Detection of BAs

The detection of BAs was carried out using TLC as described by Latorre-Moratalla et al. [49] with slight modifications. Briefly, EFEL7002 was sub-cultured four times in MRS medium containing 6% (w/v) NaCl and 0.1% of the amino acid precursors (w/v) (L-tyrosine freebase, L-histidine monochlorohydrate, L-ornithine monochlorohydrate, L-tryptophan, L-lysine monochlorohydrate, L-phenylalanine, and L-arginine (Sigma-Aldrich, St. Louis, MO, USA)) at 37 °C for 24 h. Then, the bacterial culture was centrifuged at 10,000×g at 4 °C for 10 min, and the supernatant was filtered using a 0.22 μm microfilter membrane (Polypropylene, Whatman, Kent, UK). The BAs produced were detected by a UV lamp in TLC (TLC silica gel 60G F_254_; Merck, Darmstadt, Germany) by neutralizing the sample with NaHCO_3_ and derivatizing it with dansyl chloride. A standard solution containing eight BAs (tyramine, histamine, phenylethylamine, tryptamine, putrescine, cadaverine, spermine, and spermidine) was used as the detection control.

### Hemolytic activity

The hemolytic activity of EFEL7002 was examined according to the method described by Ryu and Chang [50]. EFEL7002 was inoculated onto a BHI agar plate supplemented with 7% horse blood (MB CELL, Seoul, South Korea) and incubated at 37 °C for 24 h under anaerobic conditions. After incubation, hemolytic activity was determined and compared with that of *Listeria monocytogenes* as a positive control.

### Acid and bile salt tolerance

Tolerance to synthetic gastrointestinal juice was tested using the method described by Conway et al. [51]. EFEL7002 was cultured overnight in MRS medium containing 6% NaCl and harvested by centrifugation at 6000×g for 10 min. Cells were washed three times with phosphate-buffered saline (PBS, pH 7.2) and resuspended in an equal volume of PBS adjusted to pH 3.0 and pH 2.5 with HCl or in an equal volume of PBS containing 0.3% (w/v) bile salt (Sigma-Aldrich). After 0, 90, and 180 min incubation at 37 °C, tolerance was evaluated by spreading cells on MRS agar medium containing 6% NaCl and counting the number of viable cells after incubation at 37 °C for 48 h.

### Adhesion to intestinal epithelial cells

The capacity to adhere to intestinal epithelial cells was evaluated according to the method described by Messaoudi et al. [52]. The human colonic epithelial cell line Caco-2, was obtained from the Korean Cell Line Bank (Seoul, South Korea) and cultured in Dulbecco’s modified Eagle’s medium (DMEM; Hyclone, Logan, UT, USA) supplemented with 10% fetal bovine serum (FBS; Hyclone), 1% 10,000 U/mL penicillin, and 1% 10 mg/mL streptomycin (Hyclone) in 0.85% NaCl. Caco-2 cells were seeded in 24-well tissue culture plates (2 cm^2^/well) at a density of 4.7 × 10^5^ cells/well, and at 80% confluency the media was replaced with DMEM without antibiotics. After cultivation of EFEL7002 in MRS medium containing 6% NaCl, bacterial cells were recovered by centrifugation, washed twice with PBS (pH 7.4), and resuspended in DMEM without serum and antibiotics at a concentration of 1 × 10^8^ CFU/mL. Then, bacterial cells were applied on the Caco-2 cell monolayer (1 × 10^8^ CFU/well) and incubated at 37 °C in a 5% CO_2_ incubator for 2 h. After incubation, the non-adherent bacteria were removed by washing twice with PBS. Cells with adherent bacteria were treated with a detachment solution containing 0.1% Triton X-100 and 0.1% trypsin-EDTA (Sigma-Aldrich) for 15 min. To calculate the number of adherent bacteria, the suspensions of the detached cells were plated onto MRS agar medium containing 6% NaCl after being appropriately diluted and then incubated at 37 °C for 48 h following which the number of bacteria cells were counted.

### Protective effects on H_2_O_2_-induced intestinal permeability

Intestinal permeability measurement in the epithelial barrier model was conducted using the method described by Zaylaa et al. [53] and Miyauchi et al. [54]. Caco-2 cells were seeded on 12 well Transwell® inserts (polyester membrane with 0.4 μm pore size, 12 mm diameter; Costar, Corning Life Science, Corning, NY, USA) at a density of 5 × 10^4^ cells per well. The medium was changed every 2 days until confluent when the optimal TEER value (≥ 200 Ω·cm^2^) was reached. TEER values were measured every 2 days using Millicell-ERS (Millipore, Burlington, MA, USA). Cells were then incubated in DMEM without antibiotics before experiments and treated with bacteria (5 × 10^7^ CFU/well) 30 min prior to H_2_O_2_ treatment (100 μM), to both the upper and lower chambers. TEER was measured every 30 min for 120 min after sensitization. The results were expressed as %TEER compared with the initial TEER value at T_0_ (before the addition of H_2_O_2_) for each insert using the formula: TEER (Ω·cm^2^) / initial TEER (Ω·cm^2^) × 100 (%). FITC-dextran (100 μg/mL) was then added to the upper chamber and incubated in the dark for 4 h at room temperature. Aliquots (100 μL) of the samples were obtained from the lower chamber of each well and transferred to a black 96-well opaque plate. Fluorescence intensity was determined using a fluorescence spectrometer (LS55, Perkin Elmer Instruments, Waltham, USA) at excitation and emission wavelengths of 485 nm and 535 nm, respectively.

### Antioxidant activity

The antioxidant activity of the three fractions of bacterial cells was measured by DPPH scavenging ability, as described by Das and Goyal [55]. For preparation of intact cells, cell-free extracts, and cell-free culture supernatants, bacteria were pre-cultured for 12 h, the main culture was performed for 12 h, and optical density at 600 nm was adjusted to 1.0 (5 × 10^8^ cells/mL). The bacterial cells were washed twice and resuspended in 0.85% saline solution to isolate intact cells. For cell-free extracts, sonication was performed using a sonicator (VP-050 N; Taitec Corp., Saitama, Japan) for 10 min (pulse 5 s on/5 s off at 35% amplitude), and the cell residue was removed by centrifugation at 10,000×g at 4 °C for 5 min. For cell-free culture supernatants, bacterial cultures were centrifuged at 10,000×g at 4 °C for 10 min, and the supernatant was neutralized (pH 7.0) with 1 M NaOH and passed through 0.22 μm microfilter membrane (Whatman) to remove remaining cells. To determine antioxidant activity, 100 μL of the bacterial sample or water (negative control) was mixed thoroughly with 100 μL of 0.4 mmol/L ethanoic DPPH solution and incubated at 37 °C in the dark for 30 min. Absorbance of the mixture was measured at 517 nm using a microplate reader (BioTek, Winooski, VT, USA).

### Nitric oxide assay

The NO inhibitory activities of the EFEL7002 were determined in LPS-induced RAW 264.7 cells using the Griess reaction as described by Yu et al. [56]. For the preparation of heat-killed bacterial cells, optical density at 600 nm was adjusted to 1.0 (5 × 10^8^ cells/mL). After centrifugation at 10,000×g for 5 min, cell pellets were washed twice with PBS (pH 7.2) and resuspended in the same volume of DMEM. Next, bacterial cells were heated at 95 °C for 30 min. Lysates were prepared by sonication (pulse 5 s on/5 s off at 35% amplitude) using a sonicator (VP-050 N; Taitec Corp.) for 10 min, and the cell residue was removed by centrifugation at 10,000×g at 4 °C for 5 min. The supernatant was filtered using a 0.22 μm microfilter membrane (Whatman) to remove remaining cells. The murine macrophage cell line RAW 264.7 was maintained in DMEM supplemented with 10% FBS (Hyclone) and 1% penicillin-streptomycin (Hyclone) at 37 °C in a humidified 5% CO_2_ incubator. Cells were subcultured at 80–90% of confluence. Cells (2 × 10^5^ cells /well) were seeded in 96-well plates and incubated for 24 h. Cells were treated with 1 μg/mL LPS, followed by addition of heat-killed cells and lysates (5 × 10^7^ cells/well) for 24 h. After incubation, the culture supernatant from each well was mixed with an equal volume of Griess reagent (Sigma-Aldrich) and incubated in the dark for 15 min at 25 °C. Absorbance was measured at 540 nm using microplate reader (Bio-Tek). Nitrite concentration was calculated using dilutions of sodium nitrite as the standard and fresh culture medium was used as the blank control.

### Cytokine gene expression

The effect of EFEL7002 on the expression of pro-inflammatory cytokines was assessed in LPS-induced RAW 264.7 cells using RT-qPCR. RAW264.7 cells (1 × 10^6^ cells/well) were seeded in 6-well plates and incubated for 24 h. Cells were treated with 1 μg/mL LPS with or without heat-killed cells and lysates of EFEL7002. After incubation, the cells were washed twice with sterile PBS (pH 7.2), and total RNA was extracted using TRIzol RNA isolation reagent (Invitrogen, Waltham, MA, USA) according to the manufacturer’s protocol. RNA (200 ng/μL) was reverse-transcribed into cDNA using the LeGene Express 1st Strand cDNA Synthesis System Kit (LeGene Biosciences, San Diego, CA, USA). Real-time PCR was performed using a CFX96 real-time PCR system (Bio-Rad, Hercules, CA, USA). The reaction mixtures contained synthesized cDNA, 10 pmol of specific primers, and a Accupower® 2× Greenstar qPCR Master Mix (Bioneer, Daejeon, Korea). The reaction conditions were as follows: initial denaturation at 95 °C for 5 min; followed by 40 cycles at denaturation at 95 °C for 15 s, annealing and extension at 60 °C for 30 s; and a final extension at 60 °C for 30 s. Single-product amplification was verified by melting curve analysis. The results were analyzed after normalization with GAPDH as the reference gene. The relative expression levels of target genes were calculated using the ΔΔCt method described by Livak and Schmittgen [57]. The primer sequences used in the study are listed in Table 2.

**Table 2:** Inflammatory**-**specific primer sequences

Gene	Forward primer (5′-3′)	Reverse primer (5′-3′)
GAPDH	TTGTCTCCTGCGACTTCAACA	GCTGTAGCCGTATTCATTGTCATA
iNOS	ACCATGGAGCATCCCAAGTA	CCATGTACCAACCATTGAAGG
COX-2	AGCATTCATTCCTCTACATAAGC	GTAACAACACTCACATATTCATACAT
IL-6	AGGATACCACTCCCAACAGACCT	CAAGCGCATCATCGTTGTTGTTCATAC
IL-1β	GTTGACGGACCCCAAAAGAT	CACACACCAGCAGGTTATCA
IL-10	GGACAACATACTGCTAACCGACTC	AAAATCACTCTTCACCTGCTCCAC

### Microbial and biochemical characterization

The biochemical characteristics of EFEL7002 were analyzed using API CHL (BioMériux Co., Marcy-l’Étoile, France) according to the manufacturer’s instructions. The results were analyzed using the identification program ApiWeb software database. Type strain results were obtained from the Bacterial Diversity Metadatabase (http:// bacdive.dsmz.de; Accessed 15 June, 2021). For analysis of fermentation profile, EFEL7002 was cultured in MRS medium containing NaCl (0, 6, 12, 18% (w/v)) at 37 °C. The optical density at 600 nm and pH change were measured. For morphological observation, EFEL7002 cell pellet was treated with 2.5% glutaraldehyde solution (Sigma-Aldrich) and 1% osmium (Sigma-Aldrich) and dehydrated with absolute ethanol by concentrations. The dried samples were observed using scanning electron microscopy (SEM; ULTRA PLUS, Zeiss, Oberkochen, Germany).

### Statistical analysis

All data are presented as the mean ± standard deviation (SD). Statistical analysis was performed using the SPSS software version 22 (IBM, Armonk, NY, USA). The independent t-test was used to analyze the differences between two groups, and one-way analysis of variance (ANOVA) was used to analyze the differences between multiple groups using Duncan’s method.

## Supplementary Information

**Additional file 1.**

## Data Availability

The datasets used and analyzed within the current study are available from the NCBI website. The 16S rRNA sequence of the *T. halophilus* EFEL7002 strain was deposited with the NCBI/Genbank under accession number OM865764 (https://www.ncbi.nlm.nih.gov/nuccore/OM865764.1).

## Abbreviations

*T. halophilus* EFEL7002, EFEL7002: *Tetragenococcus halophilus* EFEL7002
BA: Biogenic amine
LAB: Lactic acid bacteria
SEM: Scanning Electron Microscope
TLC: Thin-layer chromatography
LGG: *Lacticaseibacillus rhamnosus* GG
WCFS1: *Lactiplantibacillus plantarum* WCFS1
KCLB: Korean Cell Line Bank
TEER: Transepithelial electrical resistance
CFE: Cell free extracts
CFS: Cell free supernatants
NO: Nitric oxide
LPS: Lipopolysaccharide
RT-qPCR: Quantitative real time polymerase chain reaction
IBD: Inflammatory bowel disease
PGs: Proinflammatory prostaglandins
IL-6: Interleukin 6
IL-1β: Interleukin 1β
IL-10: Interleukin 10
IL-12: Interleukin 12
SD: Standard deviation
ANOVA: One-way analysis of variance
