# Supplementary material for: Probiotic potential of Tetragenococcus halophilus EFEL7002 isolated from Korean soy Meju
Source: BMC Microbiol. 2022 Jun 6;22:149. doi: 10.1186/s12866-022-02561-7 (PMC9169274; doi:10.1186/s12866-022-02561-7)
Supplement: Supplementary file 1 — Additional file 1. [file 12866_2022_2561_MOESM1_ESM.pptx]

## Slide 1
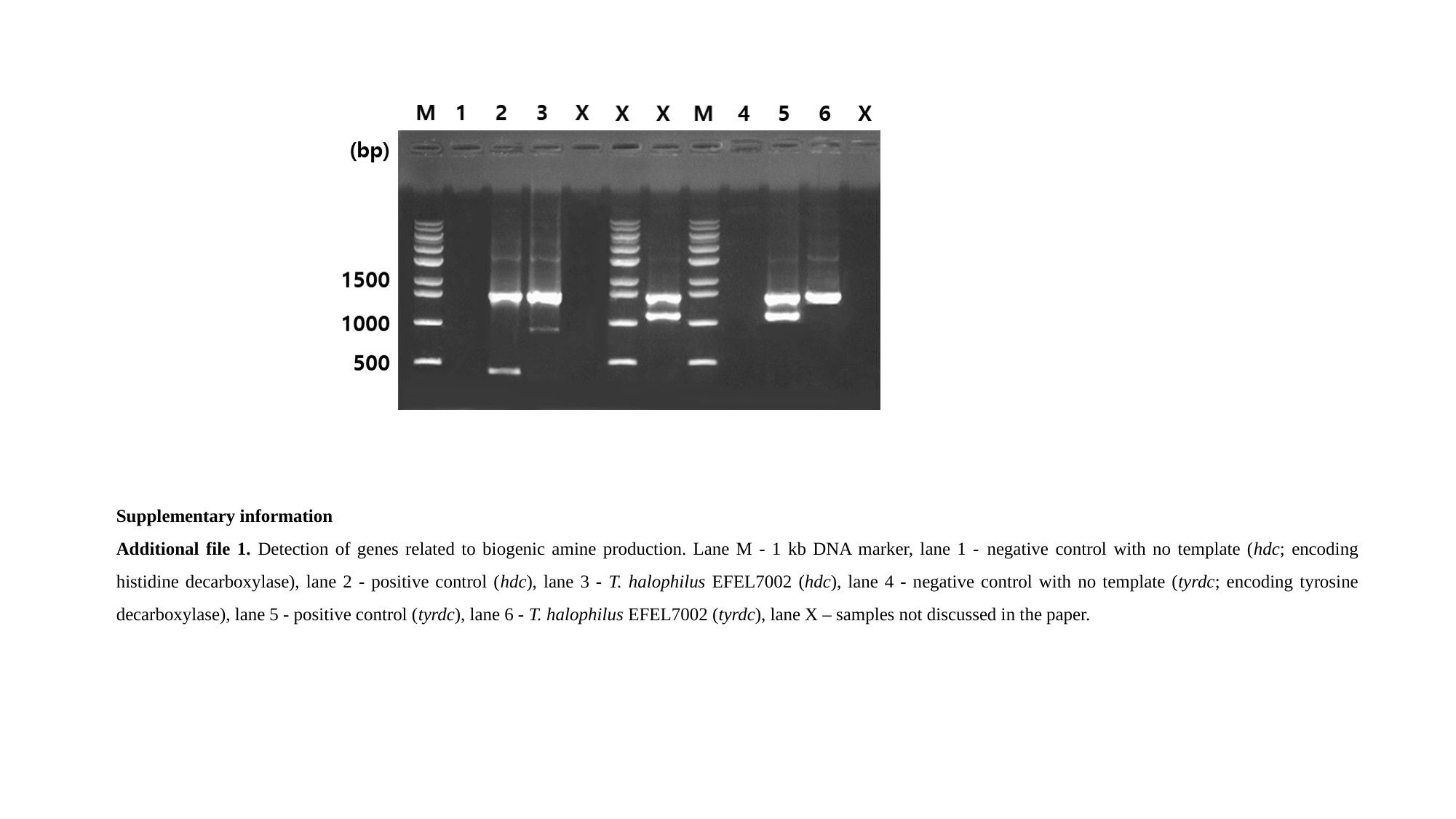

Supplementary information
Additional file 1. Detection of genes related to biogenic amine production. Lane M - 1 kb DNA marker, lane 1 - negative control with no template (hdc; encoding histidine decarboxylase), lane 2 - positive control (hdc), lane 3 - T. halophilus EFEL7002 (hdc), lane 4 - negative control with no template (tyrdc; encoding tyrosine decarboxylase), lane 5 - positive control (tyrdc), lane 6 - T. halophilus EFEL7002 (tyrdc), lane X – samples not discussed in the paper.
